# Endogenous endophthalmitis: diagnosis, management, and prognosis

**DOI:** 10.1186/s12348-015-0063-y

**Published:** 2015-11-03

**Authors:** Mohammad Ali Sadiq, Muhammad Hassan, Aniruddha Agarwal, Salman Sarwar, Shafak Toufeeq, Mohamed K. Soliman, Mostafa Hanout, Yasir Jamal Sepah, Diana V. Do, Quan Dong Nguyen

**Affiliations:** Ocular Imaging Research and Reading Center, Stanley M. Truhlsen Eye Institute, University of Nebraska Medical Center, 3902 Leavenworth Street, Omaha, NE 68105 USA; Department of Ophthalmology, Assiut University Hospital, Assiut University, Assiut, Egypt

**Keywords:** Endophthalmitis, Endogenous, Bacterial, Fungal, Review, Metastatic

## Abstract

Endogenous endophthalmitis is an ophthalmic emergency that can have severe sight-threatening complications. It is often a diagnostic challenge because it can manifest at any age and is associated with a number of underlying predisposing factors. Microorganisms associated with this condition vary along a broad spectrum. Depending upon the severity of the disease, both medical and surgical interventions may be employed. Due to rarity of the disease, there are no guidelines in literature for optimal management of these patients. In this review, treatment guidelines based on clinical data and microorganism profile have been proposed.

## Review

### Introduction

Intraocular infection affecting the inner coats of the eye associated with significant, progressive vitreous inflammation is termed as endophthalmitis [1–4]. Endophthalmitis is an ophthalmic emergency that can result in devastating ocular and systemic complications. The most common route of entry of infective organisms is through an external wound of entry, such as trauma, surgery, or infected cornea. These cases of endophthalmitis are termed as exogenous endophthalmitis. Endogenous endophthalmitis (EE), on the other hand, results from the hematogenous spread of microorganisms from distant foci [5–7].

EE accounts for approximately 2–8 % of all cases of endophthalmitis [2, 8–11]. Due to paucity of the disease, literature on EE mostly comprises of case series or single case reports. Unlike exogenous endophthalmitis, demographics, treatment options, and outcome measures in patients with EE have not been studied in large-scale studies.

The first case of bacterial EE has been published in 1856 [12]. Subsequently, a major review including approximately 335 cases of bacterial EE was published in 2003 [11], and the authors have recently updated their initial data by accommodating further reports [13]. However, there have been no major reviews encompassing all the infective etiologies, including both bacterial and fungal, in literature. With changing patterns of microbial disease epidemiology, re-emergence of certain infectious diseases, antibiotic susceptibility, and development of superbugs, a systematic reappraisal of EE is necessary.

### Causative organisms

The etiology of EE is multifactorial, and the list of causative organisms is extensive, with significant geographic variation. Both bacterial and fungal agents are noted in the literature as potential agents of EE in the developed world. However, fungal organisms account for the majority of the cases [9, 10]. The organisms responsible for bacterial EE differ depending on the geographic location. In the developed world, gram-positive organisms (*Streptococci* and *Staphylococci*) dominate the infection, whereas gram-negative organisms are more common in the Asian population [9, 14]. Asian studies have reported fungi as the causative organisms in approximately 11.1 to 17.54 % of total cases of EE, with the rest being attributed to bacterial causes [14, 15].

### Risk factors

EE is frequently associated with many underlying systemic risk factors [3, 5, 10, 16–23]. The most common risk factors include recent hospitalization, diabetes mellitus, urinary tract infection, immunosuppression (especially associated with underlying malignancy, neutropenia, and HIV (human immunodeficiency virus)), intravenous drug abuse (IVDA), and indwelling catheters [10].

Liver abscesses have been noted to be associated with EE, especially those caused by gram-negative rods such as *Klebsiella pneumonia* [24]. In most of these cases with *Klebsiella*, diabetes is the major underlying systemic risk factor [25, 26]. This finding is most prominently noted in the Asian population where bacterial endophthalmitis is more common [15]. Infective endocarditis (IE) is another important risk factor commonly associated with EE in the western countries [27, 28]. Various causes of transient bacteremia such as routine colonoscopy can also lead to EE [29].

According to a study assessing differences between the risk factors for mold and yeast infections, patients with mold infections were more likely to be associated with the use of chemotherapy as well as organ transplantation especially cardiac and liver transplants [16]. Similar results have been reported with molds as a common cause of EE in patients on immunosuppressive therapy for hematopoietic stem cell transplantation (HSCT) or for any hematological malignancy [30]. Patients with lung involvement by Aspergillus are at a specially increased risk for developing EE [4, 30, 31].

Neonatal endogenous endophthalmitis deserves a special mention. Unlike endophthalmitis in adults, neonatal cases are overwhelmingly as a result of an endogenous source of infection. Neonates with candidemia, bacteremia, and retinopathy of prematurity and low birth weight are at significant risk for developing EE [32–34]. According to a large cohort study, the odds of neonates with bacteremia, candidemia, and retinopathy of prematurity to develop EE are 21.11, 2.36, and 2.05, respectively (*p* < 0.0001) [32]. The causative organisms are often bacteria from *Streptococci* species, especially *S. agalactiae*, gram-negative rods like *Klebsiella* or *Pseudomonas* and fungi including *Candida* species. A recent report suggested decreasing incidence of neonatal EE in the developed world [32].

It is important to note that EE has also been reported in immunocompetent patients without underlying predisposing conditions. EE may be the first manifestation of an underlying occult systemic focus of infection, while the systemic cultures for infective organisms are still negative [35–39].

### Pathophysiology

Endogenous endophthalmitis results from metastatic spread of the organism from a primary site of infection in the setting of bacteremia or fungemia [40]. Most frequently, the organism reaches the eye through the posterior segment vasculature. The right eye is more commonly involved probably due to the more direct route through the right carotid artery [40]. Direct spread from contagious sites can also occur in cases of central nervous system infection via the optic nerve [41]. Unlike postoperative and posttraumatic endophthalmitis where tissue damage results primarily from toxins produced by the organism, it is postulated that in endogenous endophthalmitis, damage is most probably due to a septic embolus that enters the posterior segment vasculature and acts as a nidus for dissemination of the organism into the surrounding tissues after crossing the blood-ocular barrier to cause microbial proliferation and inflammatory reactions within these tissues. Infection then extends from the retina and the choroid into the vitreous cavity and thereafter to the anterior chamber of the eye [42].

### Clinical features

**Fig. 1:**
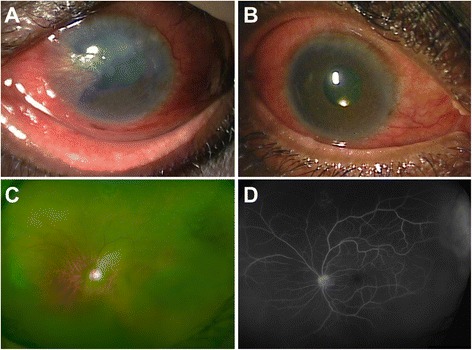
A case of bilateral tubercular endogenous endophthalmitis with scleritis. **a** Slit lamp biomicroscopy of the left eye with diffuse and circumcorneal congestion and scleral involvement. There is corneal edema and opacification superiorly. The pupil has broad-based synechiae, and the view of the posterior segment was hazy. **b** The right eye with severe congestion and ciliary injection. There was a yellow glow present (visible near the inferior pupillary border). **c** A wide-angled fundus photograph of the left eye with vitreous haze secondary to vitritis along with focal sheathing of superior vessels. The fluorescein angiography (**d**) shows presence of superior perivascular hyperfluorescence and leakage of dye in the superotemporal periphery

The diagnosis of EE may be difficult because of the variability in the clinical signs and symptoms. The organisms causing EE gain access to the internal ocular tissues through the blood-ocular barrier [43]. Due to progressive inflammation, the patients may experience decreased vision, which is the most common reason for visiting a doctor [5, 18, 37]. The other classic features include eyelid edema, conjunctival injection, circumcorneal congestion, pain, photophobia, and the presence of floaters [5]. Anterior chamber inflammation with hypopyon, absent red reflex, vitreous cells, and haze may also occur [21, 28]. These findings of anterior chamber involvement are more common in bacterial causes of EE [6]. There may be a poor view of the fundus due to the presence of exudates and vitreous haze. Other findings include corneal edema, presence of iris nodules, and pupillary distortion secondary to synechiae formation [44, 45]. Bilateral involvement can also occur. Causative organisms such as *Mycobacterium tuberculosis* can present with bilateral endogenous endophthalmitis and scleral inflammation (Fig. 1).

**Fig. 2:**
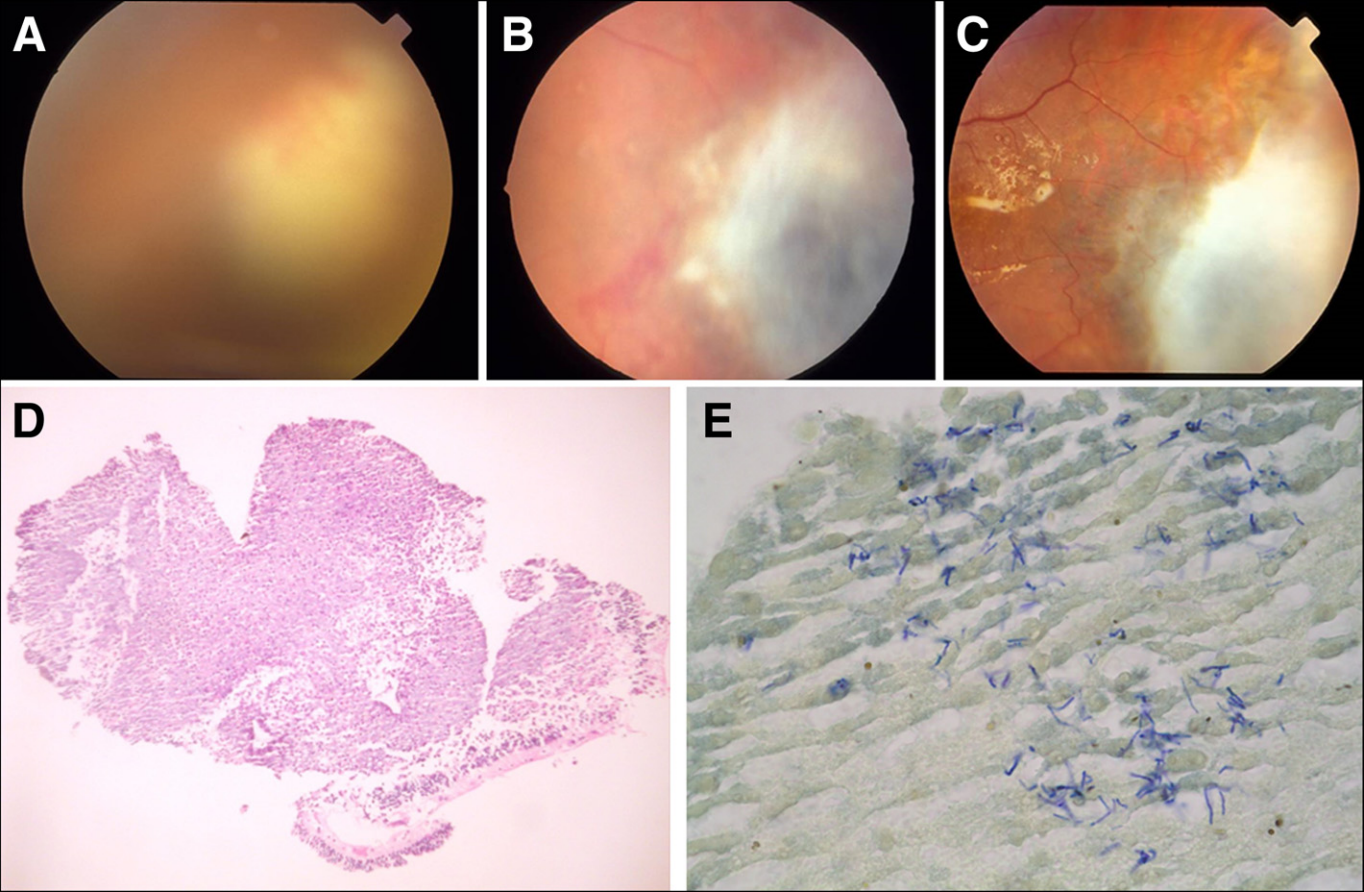
Fundus photograph of a 78-year-old male (**a**) with a yellow white mass in the temporal paramacular region with some superficial hemorrhages suggestive of a choroidal abscess. The patient was diagnosed with *Nocardia* endophthalmitis based on retinal aspirates (**d**, **e**). **b** Fundus photograph taken at 3 weeks following intravenous trimethoprim-sulfamethoxazole therapy. There was a marked resolution of the lesion and improvement in media clarity at month 3 (**c**). **d** Hematoxylin-eosin staining (×20) of the retinal aspirate. **e** Gram-positive branching rods of *Nocardia* species (×40)

The hallmark of EE is significant involvement of the vitreous cavity. Vitreous involvement by *Candida* can present as vitritis or fluffy white retinal lesions extending into the vitreous [43]. *Aspergillus* can present as yellow/white lesions which can be focal or diffuse [4, 20, 37] (Fig. 2). If the media clarity permits, retinal hemorrhage and cotton wool spots may be visualized on examination [37]. Severe vitreal involvement in bacterial EE can present with a sub-retinal and choroidal abscess [46]. Methicillin-resistant *Staphylococcus aureus* (MRSA) associated endophthalmitis is associated with high rates of retinal detachment especially when the time period between onset of symptoms and presentation is delayed by more than 2 weeks [3]. Other non-specific findings can include flame-shaped hemorrhages, Roth spots and cotton wool spots [6, 45].

Clinical findings in EE can be subdivided into three categories to aid the ophthalmologist to rule in the diagnosis. Positive signs are strongly suggestive of endogenous endophthalmitis, whereas probable signs are non-specific but could be present in a case of EE. Table 1 provides a list of clinical signs associated with EE.

**Table 1:** Ocular signs suggestive of endogenous endophthalmitis [13, 42]

Positive	Possible	Probable
Uveal tissue abscesses	Hypopyon ≤ 1.5 mm	Conjunctival injection/chemosis
Hypopyon ≥ 1.5 mm	Vitreous haze but no visible exudates	Anterior chamber inflammation but no hypopyon
Vitreous exudates	Non-necrotizing, focal, discrete chorioretinal lesions	Absence of vitreous haze
Visible arteriolar septic emboli	Optic neuritis	Lid edema
Necrotizing retinitis	Intra-retinal hemorrhages	Fever
Perivascular hemorrhages with inflammatory infiltrate	Neonate with white reflex^a^	
Panophthalmitis	Scleritis	
Corneal infiltrates or ulcer		

Varying combination of symptoms may be present

^a^In a neonate presenting with white reflex, endogenous endophthalmitis can be considered in the differential diagnosis

Visual acuity, as explained above, can be variably affected at the time of presentation, but is generally used as an outcome measure along with a dilated funduscopic examination to follow up the patient after starting treatment. A relative afferent pupillary defect (RAPD) can also be present and can guide the need for a vitrectomy [26].

A large study was conducted to assess the involvement of eyes in patients with candidemia. A total of 370 patients were enrolled; among them, 60 (16.2 %) patients were found to have ocular manifestations on fundoscopic examination. Among these 60 subjects with ocular involvement, 6 patients were diagnosed with EE [43]. In approximately 18 % of the patients, new lesions were seen after an initial negative funduscopic examination. This led to a hypothesis that there is a significant time delay between seeding and development of visible retinal lesions; therefore, patients may have a normal retinal exam initially.

In order to classify the severity of ocular involvement in EE, numerous attempts have been made to classify the disease. However, there is no unifying broadly accepted classification for EE available till date. Ishibashi et al. and Petit et al. have previously proposed clinical classifications of fungal EE [47, 48].

### Diagnosis

The diagnosis of EE requires a high index of suspicion with presence of one of the above mentioned systemic risk factors and/or presence of characteristic ocular findings on detailed ophthalmoscopic examination (Table 1) [49]. However a clinical diagnosis of EE is always difficult as it has a high false negative rate for EE [5, 49]. Multiple clinic visits may be required to confirm the diagnosis. It is also important to note that the presence of EE is generally not among the major concerns in patients with life-threatening invasive fungal diseases or sepsis secondary to a bacterial etiology [50], and hence the diagnosis of EE may be delayed with other morbidities being managed acutely.

To confirm the presence of a specific etiology, vitreous aspiration and diagnostic vitrectomy followed by a culture and histological examination are commonly used [16, 43, 51]. The need for a diagnostic vitrectomy is dependent on the clinician’s judgment. Vitrectomy has a higher diagnostic yield for culture (92 %) compared to a vitreous aspirate (44 %) as shown by Lingappan et al. [5]. Similar results were obtained in another study with needle biopsy negative cases growing organisms on culture following vitrectomy [52]. The study showed that vitreous samples during vitrectomy were taken near the retinal surface, which can potentially explain the lower yield of needle biopsy as early or localized infection located near the retinal surface might be missed by a needle biopsy [16].

Another emerging technique is the use of real-time polymerase chain reaction (RT-PCR) of aqueous and vitreous samples for detection of the etiology of EE. Sugita et al. reported excellent sensitivity as well as specificity of RT-PCR for detection of fungi [53]. In the same study, PCR was able to detect causative fungi in 5 culture negative specimens. This technique has the advantage of rapid diagnosis (within 90 min), better detection than cultures as well as no fear of contamination of culture samples yielding false positive results [10, 54, 55]. Somya et al. in their study demonstrated increased sensitivity of PCR over culture [56]. PCR-Based techniques can be used to rule out the presence of pathogens with confidence, which is a unique advantage of this methodology. This diagnostic tool promises to be useful in the management of patients with endophthalmitis, especially in samples that are culture negative [57]. However, a potential disadvantage of this diagnostic technique is the inability to determine antibiotic susceptibility [21].

The most reliable way of diagnosing systemic infection is blood culture. Blood must be drawn on three consecutive days using sterile precautions. Previous large series have shown higher rates of positivity following blood culture as compared to vitreous aspirate possibly due to larger volume sampled. It is also important to culture other extra ocular sites to identify the possible nidus of infection and guide systemic therapy accordingly, for example, urine cultures. Confirmatory identification of extra ocular sources of infection are reported in 21–100 % of cases in the literature [5, 18, 21]. Identification of these infectious foci is particularly important in cases where vitreous cultures are negative [15].

**Fig. 3:**
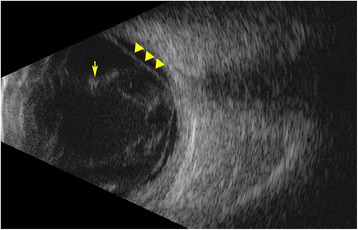
Ultrasound B-scan of a patient diagnosed with endogenous bacterial endophthalmitis following septic arthritis. There is presence of dense, hyper-reflective echoes in the vitreous cavity suggestive of exudates (*yellow arrow*). The membrane-like echo in the scan marked by *yellow triangles* suggests presence of a total retinal detachment

Imaging of ocular tissues is an important means to diagnose intraocular infection. Presence of exudates in the vitreous cavity can present as echoes in the ultrasound B-scan of the eye. Patients with EE can present with abscesses in the choroid (Fig. 2). These can be detected as dome-shaped lesions arising from the choroid on B-scan. Complications of EE, including retinal detachment may be difficult to assess clinically. In such situations, ultrasound B-scan can help in identification of retinal detachment (Fig. 3). Optical coherence tomography (OCT) has also been used as an imaging modality in patients with EE where it helps in localizing the pathology within the retinal layers as well as sub-retinal space [58, 59]. It can demonstrate sub-retinal exudates with elevation of retinal pigment epithelium, intra-retinal lesions with or without extrusion into the vitreous, choroidal thickening, and posterior vitreous cells [59, 60].

### Treatment

**Fig. 4:**
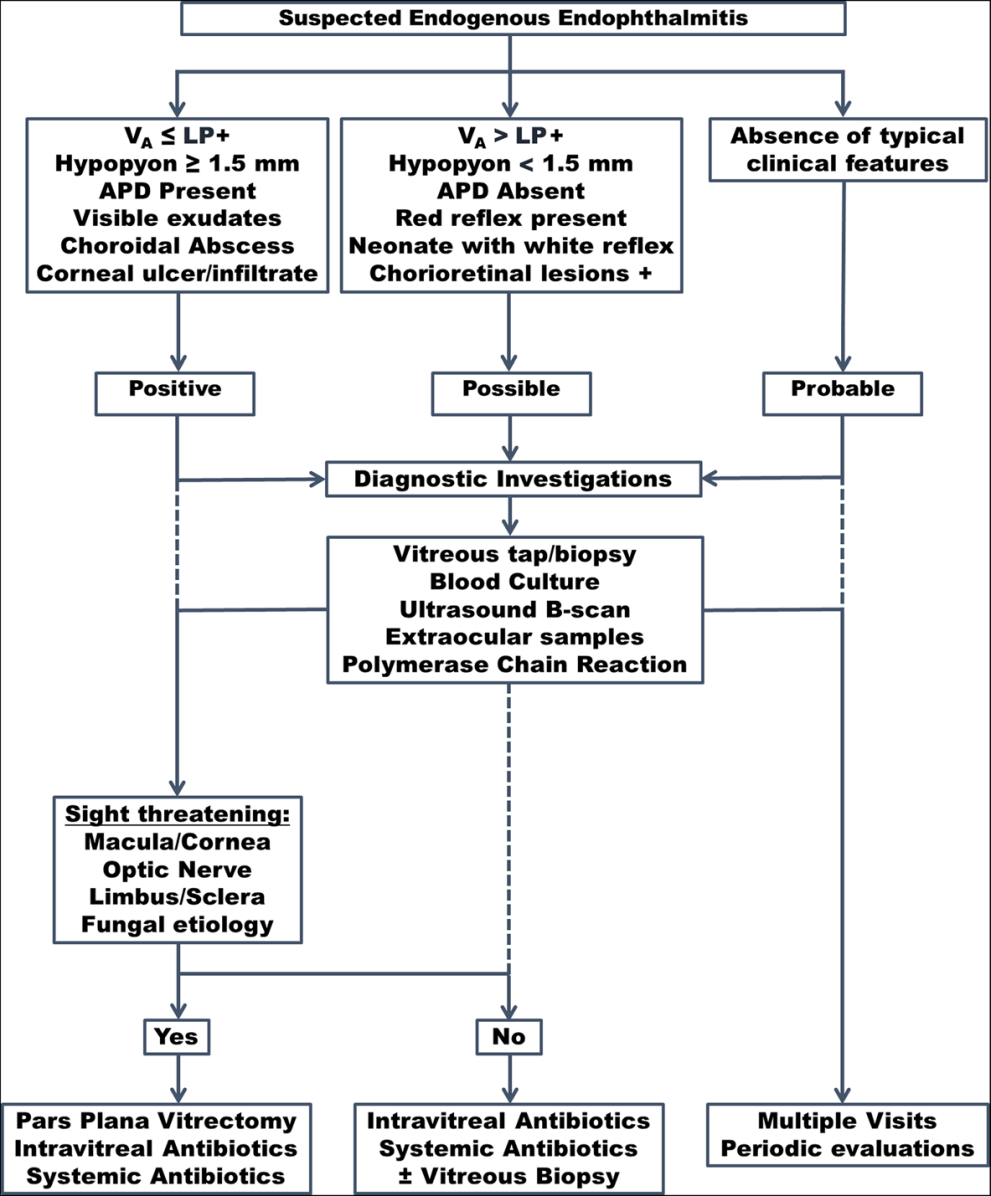
A proposed management of patients with endogenous endophthalmitis. Signs such as poor visual acuity (≤ perception of light), large hypopyon, and choroidal abscess make the diagnosis of endophthalmitis very likely. In a neonate with white reflex, endophthalmitis (along with other considerations such as malignancy) must be kept as a possibility in the differential diagnosis. Sight-threatening lesions involving the fovea, optic nerve head, cornea, limbus, or sclera may require prompt surgical management. *APD* afferent pupillary defect, *V*_*A*_ visual acuity, *LP* light perception

As an ophthalmological emergency, prompt management is required for any patient with suspected EE. Approach to management of such a patient involves assessment of degree of ocular involvement, identification of the causative organism, and the underlying source of infection and then treatment of both the endophthalmitis and the underlying systemic infection. Summary of the steps in the diagnosis and management of EE are illustrated in Fig. 4.

### Bacterial endogenous endophthalmitis

#### Systemic therapy

Treatment of the underlying source of bacteremia is necessary with systemic antibiotics. Systemic antibacterial therapy should be initiated after blood cultures have been obtained. However, treatment with systemic antibiotics tailored to systemic infection alone is not sufficient, and most patients with severe endogenous bacterial endophthalmitis may require intravitreal antibiotics. In addition, pars plana vitrectomy (PPV) may also be needed for the treatment of endogenous bacterial endophthalmitis.

#### Local therapy

Cultures of vitreous obtained by needle aspiration or vitrectomy are indicated as soon as infectious endophthalmitis is suspected. Timing of administration of the intravitreal antibiotics has not been officially established; however, Yonekawa et al. showed that early administration, i.e., within 24 h, was associated with a favorable outcome [28]. Treatment is first initiated with empirical intravitreal antibiotics that provide a cover for both gram-positive and gram-negative organisms, when the etiology is unknown. These include vancomycin 1 mg/0.1 ml plus either ceftazidime 2.25 mg/0.1 ml or amikacin 0.4 mg/0.1 ml [12, 26, 44]. For gram-positive infections, vancomycin is the primary drug used because of emergence of many cases of methicillin-resistant organisms [28]. However, recently, there have been reports of gram-positive cases resistant to vancomycin [61]. Khera et al. reported seven cases of EE caused by vancomycin-resistant bacteria [62].

There are multiple therapeutic agents that can be used for gram-negative infections. The most commonly used drug to provide gram-negative coverage is ceftazidime (2.25 mg/0.1 ml) or amikacin (400 μg/0.1 ml) [14, 28, 63]. Fluoroquinolones also have good gram-positive and gram-negative coverages, especially the fourth generation fluoroquinolones [61]. However, recently, resistance against fluoroquinolones is on a rapid rise [64–66]. Antibiotics can be tailored further once the organism is identified and susceptibility pattern is known from vitreous and blood cultures. Table 2 lists the most commonly used intravitreal antibiotics.

**Table 2:** Commonly used intravitreal antibacterial drugs used for pharmacotherapy of bacterial endogenous endophthalmitis

Drug	Intravitreal dose	Reference
A. Gram-positive bacteria (including VSSA)		
Vancomycin	1 mg/0.1 ml	[12, 26, 44]
Cefazolin	2.25 mg/0.1 ml	
B. Gram-positive bacteria—VRSA		
Daptomycin	200 μg/0.1 ml	[27]
Quinupristin/dalfopristin	0.4 mg/0.1 ml	[62]
C. Gram-negative bacteria		
Ceftazidime	2.25 mg/0.1 ml	[12, 26]
Amikacin	0.4 mg/0.1 ml	[89]

*VSSA* vancomycin sensitive staphylococcus aureus, *VRSA* vancomycin resistant staphylococcus aureus

Early diagnosis and treatment is essential for a better prognosis. However, patients with endogenous bacterial endophthalmitis may have a delayed diagnosis which may lead to poor prognoses [3, 11, 67].

Two important groups that need special attention while administering antibiotics are pregnant and breastfeeding women. Penicillins, cephalosporins, and erythromycin are among the mainline agents in these groups due to good safety profiles [29]. Fluoroquinolones have been associated with abnormalities of developing cartilage in animal studies [68]. Even though there have been no reports of such cases during human pregnancies, it is recommended to use fluoroquinolones only when other safer alternatives are not available despite their good vitreous penetration [29, 68, 69].

### Fungal endogenous endophthalmitis

#### Endogenous *Candida* endophthalmitis

For severe vitritis, the best approach appears to be vitrectomy accompanied by intravitreal injection of amphotericin or voriconazole and systemic antifungal therapy [70, 71]. The dose of amphotericin B (AMB) deoxycholate for intravitreal injection is 5 to 10 mcg in 0.1 ml sterile water or dextrose. This dose appears to be safe and can be repeated after intervals of 48 h or more if there is evidence of persistent intraocular infection. Systemic administration of AMB is associated with dose-limiting nephrotoxicity, hypotension, arrhythmias, and infusion-related fever and chills (“shake and bake”) [8]. Voriconazole is a newer agent in the armory of drugs used to treat ocular fungal infections. It achieves an excellent intravitreal concentration after oral or intravenous administration [36]. The usual dose of voriconazole is 100–200 mcg in 0.1 ml sterile water. This dose achieves a final concentration of about 25–50 mcg/ml in the vitreous [72].

Among the azoles, the recommended dose of fluconazole according to the Infectious Disease Society of America (IDSA) guidelines for *Candida* endophthalmitis is 400–800 mg daily [73]. Fluconazole is also a broad spectrum agent with a better side effect profile than AMB. Therefore, it has been used in place of AMB as the first-line agent against endogenous fungal endophthalmitis (EFE) as shown by Hamada et al. [74]. IDSA recommended the use of amphotericin B along with flucytosine for *Candida* endophthalmitis. Alternatively, fluconazole can be used as well. For severe cases of endophthalmitis or vitritis, the adjunctive use of vitrectomy is recommended [73].

The duration of systemic antifungal therapy is a minimum of 6 weeks but the length of therapy depends upon the resolution of ocular lesions. With severe involvement, usually a longer duration of therapy may be required.

#### Other endogenous fungal endophthalmitis

Treatment in immunocompromised patients includes systemic antifungal therapy (e.g., amphotericin or voriconazole). If the patient is able to tolerate surgery, vitrectomy and removal of intraocular lens should be performed followed by intravitreal antifungal therapy using amphotericin or voriconazole. However, if the patient cannot tolerate surgery, intravitreal injection with amphotericin or voriconazole should be administered initially and repeated as needed. Voriconazole has been used to treat fungal infections resistant to fluconazole and amphotericin B [75]. In an in vitro study, voriconazole showed 100 % activity against *Aspergillus* species, *Paecilomyces* species, and *Fusarium* species [76]. Other reports also stated successful treatment of *Fusarium* and *Aspergillus* endophthalmitis using voriconazole [77, 78].

IDSA guidelines for the treatment of *Aspergillus* endophthalmitis recommend the use of IV amphotericin B with addition of intravitreal amphotericin B and pars plana vitrectomy for sight-threatening cases [79]. The recommended alternate therapy is systemic or intravitreal voriconazole. Table 3 summarizes the role of antifungal agents along with their sensitivity profiles.

**Table 3:** Commonly used intravitreal antifungal drugs employed for pharmacotherapy of fungal endogenous endophthalmitis along with their sensitivity

Drug	Intravitreal dose	Systemic dose	*Candida*	*Aspergillus*	Others
A. Polyene					
Amphotericin B	5 μg/0.1 ml	0.5–0.7 mg/kg (IV)	++	+	
B. Imidazoles					
Miconazole	25–50 μg/0.1 ml	–	+	+	
Itraconazole	5 μg/0.05 ml	200–400 mg/day (oral)	+	+	
		200 mg/day (IV)			
Voriconazole	50–200 μg/0.1 ml	200 mg twice daily (oral)	+++	++	Fusarium +
		3–6 mg/kg (IV) twice daily			
C. Pyrimidine					
5-Flucytosine	2.25 mg/0.1 ml	25–37.5 mg/kg/day	−	+	
D. Echinocandins					
Caspofungin	–	50 mg/day	+	+	

*IV* intravenous

### Pars plana vitrectomy

PPV is a commonly used modality in the treatment of EE. It is recommended for severe and sight-threatening *Candida*, *Aspergillus*, or bacterial endophthalmitis [5, 73, 79]. It serves as a diagnostic as well as therapeutic purpose. It may remove a large number of organisms seeding the vitreous cavity thus lowering the disease burden [2, 5, 80]. An intravitreal injection of drugs may also be given while performing the surgery. The decision regarding vitrectomy is usually based on the clinician’s judgment. However, almost all reported cases where a therapeutic vitrectomy was performed are of patients presenting with either sight-threatening disease or of those that were irresponsive to systemic therapy [15, 17, 49, 52, 67, 80].

Zhang et al. has reported better visual outcomes in cases that underwent early vitrectomy [52]. Decision of early vitrectomy has also been associated with a decrease in incidence of retinal detachment and evisceration or enucleation [15, 81]. Sato et al. recommended the use of vitrectomy for *Candida* EE before stage IV according to Ishibashi’s classification [47]. In cases of bacterial EE, vitrectomy is generally performed when there is no response to intravitreal antibiotics within 48 h or when the eye condition continues to decline or with a worse grade of RAPD [26]. Yoon et al. and Ishii et al. suggested aggressive treatment including early vitrectomy for *Klebsiella* endophthalmitis might lead to better final outcomes [82, 83]. On the other hand, Sheu et al. found no association between the timing of vitrectomy and visual outcome in *Klebsiella* endophthalmitis [25]. However, they still suggested the use of surgical intervention, especially in patients with anterior chamber inflammation that did not respond well to intravitreal antibiotics.

### Role of corticosteroids

Currently, no clear guidelines exist regarding the use of corticosteroids in endophthalmitis. Inflammation, although essential in combating invading organisms, may end up damaging retinal structures [84]. Steroids have multiple anti-inflammatory effects which include but are not limited to decrease in leucocyte recruitment, attenuating production of various inflammatory cytokines and stabilizing membrane barriers including blood-retinal barrier [85].

Clinical studies have reported controversial results on the use of intravitreal as well as systemic steroids for endophthalmitis [2]. In two case series by Jackson et al., better visual outcomes were reported in the patients who received additional treatment with intraocular steroids [11, 13]. An interim safety analysis of a prospective multicenter randomized placebo-controlled trial of IVT dexamethasone as an adjuvant therapy for endophthalmitis did not report any safety risks associated with the use of steroids [85]. On the other hand, Shuwan lee et al. reported no significant association of the use of systemic steroids with better visual outcomes [86]. Shah et al. reported a significantly reduced likelihood of obtaining a three-line improvement in visual outcomes following the use of intravitreal steroids in patients with postoperative endophthalmitis [87].

In summary, data on the use of steroids in endophthalmitis is limited, and the results of studies are conflicting. Therefore, judicious use of steroids is recommended.

### Prognosis

In general, EE does not have a favorable prognosis and results in complete vision loss, especially if the diagnosis is missed early on and therefore treatment is delayed [21]. Zenith et al. reported that the eyes with bacterial EE had a worse outcome with more patients requiring enucleation or evisceration compared to patients with fungal EE [21]. The major risk following vitreous aspirate in patients with EE is high incidence of retinal detachment. Surgery for retinal detachment in these cases is difficult, and there is a need for long-term tamponade in such patients post vitrectomy [88].

A clinician has to maintain a very high level of suspicion when a patient with a possible risk factor presents in association with decreased vision and vitreoretinal changes on examination. Early diagnosis and treatment has been associated with 64 % of patients having visual acuity of counting fingers (CF) or better in one study for bacterial EE [28]. This is well above the percentage of patients reported with similar improvement before this study [11]. Itoh et al. also reported that early aggressive treatment can lead to good visual outcomes [89]. Early vitrectomy within 2 weeks of presentation, especially in severe cases or when suspecting a highly virulent organism, can lead to a good overall outcome [79, 82, 83, 86, 90].

Virulence of the organism plays an important role in the visual outcome [15]. *Aspergillus* and other molds cause more aggressive disease compared to yeasts and therefore carries a worse prognosis [16, 18, 30, 43, 91]. Similarly MRSA endophthalmitis has been reported to be associated with significant mortality [28]. The association of MRSA endophthalmitis with visual outcome has been variable, with some studies reporting no association while others associating it with worse visual outcome [28, 92, 93]. Connell et al. found that all the patients in their study needing enucleation were infected by *Klebsiella* [10].

In a study conducted to determine factors resulting in poor visual outcome, worse initial visual acuity and centrally located lesions were found to be associated with poor visual outcomes [81]. The same study showed that early vitrectomy prevented the development of retinal detachment. The results of another study in patients with fungal EE showed that early stages were associated with better prognosis. This underscores the importance of detecting and promptly treating the disease at early stages to preserve visual acuity [80]. According to Ang et al., the main prognostic factor in *Klebsiella* EE is the presence of hypopyon [26]. Other prognostic factors found in the same study include rapid onset of ocular symptoms, unilateral involvement, and panophthalmitis. Another study found no association between final visual acuity (log MAR values) and diabetes, causative organism, source of infection, and performance of vitrectomy [15]. However, the study did report better final visual outcomes in patients with initial visual acuity better than counting fingers.

## Conclusions

EE is an ophthalmological emergency that requires prompt diagnosis and management. Figure 4 depicts a simplified flow chart for the diagnosis and management of EE. The main challenges in the management of EE are early identification and delivering an adequate concentration of the drug in the vitreous cavity. It may be possible to overcome this challenge with direct intravitreal administration of the antibiotic.

Systemic therapy is used to treat the focus of infection causing the metastatic spread of the organism to the ocular cavity. In mild cases of EE, systemic therapy is the mainstay of treatment. However, in severe cases, systemic therapy is adjuvant to the more aggressive intravitreal administration of drugs.

PPV has a diagnostic as well as therapeutic role in the management of EE. Vitrectomy may be strongly considered as a treatment option if there is no response to systemic or local therapy within 24–48 h of presentation or if the patient has possible worsening. Visual acuity, systemic debility, etiology of infection, and ocular examination must guide the decision to intervene in such cases.

## Abbreviations

AMB: Amphotericin B
CF: Counting fingers
EE: Endogenous endophthalmitis
EFE: Endogenous fungal endophthalmitis
HIV: Human immunodeficiency virus
HSCT: Hematopoietic stem cell transplantation
IDSA: Infectious Disease Society of America
IE: Infective endocarditis
IVDA: Intravenous drug abuse
MRSA: Methicillin-resistant *Staphylococcus aureus*
OCT: Optical coherence tomography
PCR: Polymerase chain reaction
PPV: Pars plana vitrectomy
RAPD: Relative afferent pupillary defect
RT-PCR: Real-time polymerase chain reaction
